# Myeloid cell‐derived tumor necrosis factor‐alpha promotes sarcopenia and regulates muscle cell fusion with aging muscle fibers

**DOI:** 10.1111/acel.12828

**Published:** 2018-09-06

**Authors:** Ying Wang, Steven S. Welc, Michelle Wehling‐Henricks, James G. Tidball

**Affiliations:** ^1^ Molecular, Cellular and Integrative Physiology Program University of California Los Angeles California; ^2^ Department of Integrative Biology and Physiology University of California Los Angeles California; ^3^ Department of Pathology and Laboratory Medicine, David Geffen School of Medicine at UCLA University of California Los Angeles California

**Keywords:** aging, macrophage, sarcopenia, skeletal muscle, tumor necrosis factor‐α

## Abstract

Sarcopenia is age‐related muscle wasting that lacks effective therapeutic interventions. We found that systemic ablation of tumor necrosis factor‐α (*TNF‐*α) prevented sarcopenia and prevented age‐related change in muscle fiber phenotype. Furthermore, *TNF*‐α ablation reduced the number of satellite cells in aging muscle and promoted muscle cell fusion in vivo and in vitro. Because CD68+ macrophages are important sources of TNF‐α and the number of CD68+ macrophages increases in aging muscle, we tested whether macrophage‐derived TNF‐α affects myogenesis. Media conditioned by *TNF*‐α‐null macrophages increased muscle cell fusion in vitro, compared to media conditioned by wild‐type macrophages. In addition, transplantation of bone marrow cells from wild‐type mice into *TNF*‐α‐null recipients increased satellite cell numbers and reduced numbers of centrally nucleated myofibers, indicating that myeloid cell‐secreted TNF‐α reduces muscle cell fusion. Transplanting bone marrow cells from wild‐type mice into *TNF*‐α‐null recipients also increased sarcopenia, although transplantation did not restore the age‐related change in muscle fiber phenotype. Collectively, we show that myeloid cell‐derived TNF‐α contributes to muscle aging by affecting sarcopenia and muscle cell fusion with aging muscle fibers. Our findings also show that TNF‐α that is intrinsic to muscle and TNF‐α secreted by immune cells work together to influence muscle aging.

## 1 INTRODUCTION

Aging skeletal muscle undergoes a gradual decline in mass, termed “sarcopenia.” In humans, sarcopenia causes a 30%–50% reduction in muscle mass from the fourth to the eighth decade of life and muscle functional capacity declines at the rate of up to a 3% annually after age 60 (Evans & Lexell, 1995; Melton et al., 2000; Metter, Conwit, Tobin, & Fozard, 1997). Sarcopenia is also associated with decreased muscle regenerative capacity that can further exacerbate loss of function. Together, these changes lead to increased risk of falling, loss of physical independence, and increased morbidity and mortality (Landi et al., 2012; Patel et al., 2014; Xue, Walston, Fried, & Beamer, 2011). Although sarcopenia is influenced by intrinsic changes in muscle (Sousa‐Victor et al., 2014) and by extrinsic factors from other systems (Carlson & Faulkner, 1989; Fry et al., 2015), the specific mechanisms that drive sarcopenia are largely unknown.

Inflammaging, the increase in chronic low‐grade systemic inflammation that occurs during aging, is associated with sarcopenia and frailty (Jo, Lee, Park, & Kim, 2012; Visser et al., 2002) and involves an increase in resident macrophage populations within aging muscles (Wang, Wehling‐Henricks, Samengo, & Tidball, 2015). Macrophages are primary sources of inflammatory cytokines, including tumor necrosis factor‐alpha (TNF‐α), that have the potential to affect muscle mass and function. TNF‐α increases in the serum of men during aging (Léger, Derave, Bock, Hespel, & Russell, 2008) which correlates with the loss of muscle mass and strength (Greiwe, Cheng, Rubin, Yarasheski, & Semenkovich, 2001; Visser et al., 2002). Moreover, in other models of muscle atrophy that include cachexia and injury‐induced muscle atrophy, administration of exogenous TNF‐α to mice decreases muscle mass and regenerative capacity (Coletti, Moresi, Adamo, Molinaro, & Sassoon, 2005; Song, Saeman, Libero, & Wolf, 2015). Those data suggest that macrophage‐secreted TNF‐α may play an important role in muscle aging.

TNF‐α may contribute to sarcopenia by regulating the number or regenerative capacity of satellite cells. Satellite cells are specialized myogenic stem cells that are located beneath the basement membranes of muscle fibers and are required for muscle regeneration and growth (Sambasivan et al., 2011). In response to injury, satellite cells exit their quiescent state and then proliferate, differentiate, and fuse with existing myofibers to provide new myonuclei and replace or repair injured cells (Relaix & Zammit, 2012). These regenerative functions of satellite cells can be affected by TNF‐α which reduces myogenic differentiation through transcriptional activation of NF‐κB and decreases protein stability of MyoD, a transcription factor that plays a key role in regulating myoblast transition from proliferation to differentiation (Guttridge, Mayo, Madrid, Wang, & Baldwin, 2000; Langen et al., 2004). In addition, in vitro observations show that TNF‐α has bimodal effects on myogenesis, promoting myoblast proliferation at early stages of myogenesis while repressing myoblast differentiation (Chen, Jin, & Li, 2007; Langen, Schols, Kelders, Wouters, & Janssen‐Heininger, 2001; Palacios et al., 2010).

Coinciding with elevated serum concentration of TNF‐α, the myogenic capacity of satellite cells declines during aging (Conboy, Conboy, Smythe, & Rando, 2003; Sousa‐Victor et al., 2014), suggesting the unexplored possibility that TNF‐α may contribute to sarcopenia by influencing the regenerative capacity of aging satellite cells. Intriguingly, whether the age‐related impairment of satellite cell function is caused by changes intrinsic to satellite cells or by extrinsic factors is disputed. For example, previous studies of transplanted muscles showed impaired regeneration of young muscles transplanted into old animals but improved regeneration of old muscles when incorporated into young animals (Carlson & Faulkner, 1989; Roberts, McGeachie, & Grounds, 1997). Those observations indicated a role for the tissue environment extrinsic to muscle cells in regulating their regeneration. Similarly, heterochronic parabiosis experiments showed that exposure of aged satellite cells to a youthful environment could enhance the regenerative capacity of the skeletal muscle from old animals (Conboy et al., 2005). However, other investigators reported that transplanting old satellite cells into a young environment failed to restore the phenotype or myogenic capacity of satellite cells (Bernet et al., 2014; Cosgrove et al., 2014). Despite the apparent discrepancies that may be caused by different experimental approaches, these data together suggest that both intrinsic and extrinsic factors contribute to muscle aging. Interestingly, TNF‐α is expressed in both myofibers and tissue‐resident macrophages in aging muscle, suggesting that TNF‐α might serve as one of the nodes integrating intrinsic and extrinsic factors affecting sarcopenia.

In this study, we used a mouse model with systemic genetic ablation of *TNF*‐*α* (*TNF*‐*α*‐null mice) to test the hypothesis that TNF‐α regulates sarcopenia and satellite cell function. We found that ablating *TNF*‐*α* resulted in reduced sarcopenia and produced hypernucleation of aging myofibers. Furthermore, our in vitro experiments showed that TNF‐α intrinsic to satellite cells and TNF‐α secreted by macrophages both influence myogenesis. We then specifically tested the importance of myeloid‐cell‐secreted TNF‐α in vivo by performing transplantation of wild‐type bone marrow cells (BMCs) into *TNF*‐*α*‐null mice and found that myeloid‐cell‐derived TNF‐α contributed to sarcopenia and reduced muscle cell fusion, establishing the role of myeloid‐cell‐derived TNF‐α in influencing muscle aging.

## 2 RESULTS

### 2.1 Genetic ablation of *TNF‐α* prevents sarcopenia and muscle fiber type composition switch in aging muscle

**Figure 1:**
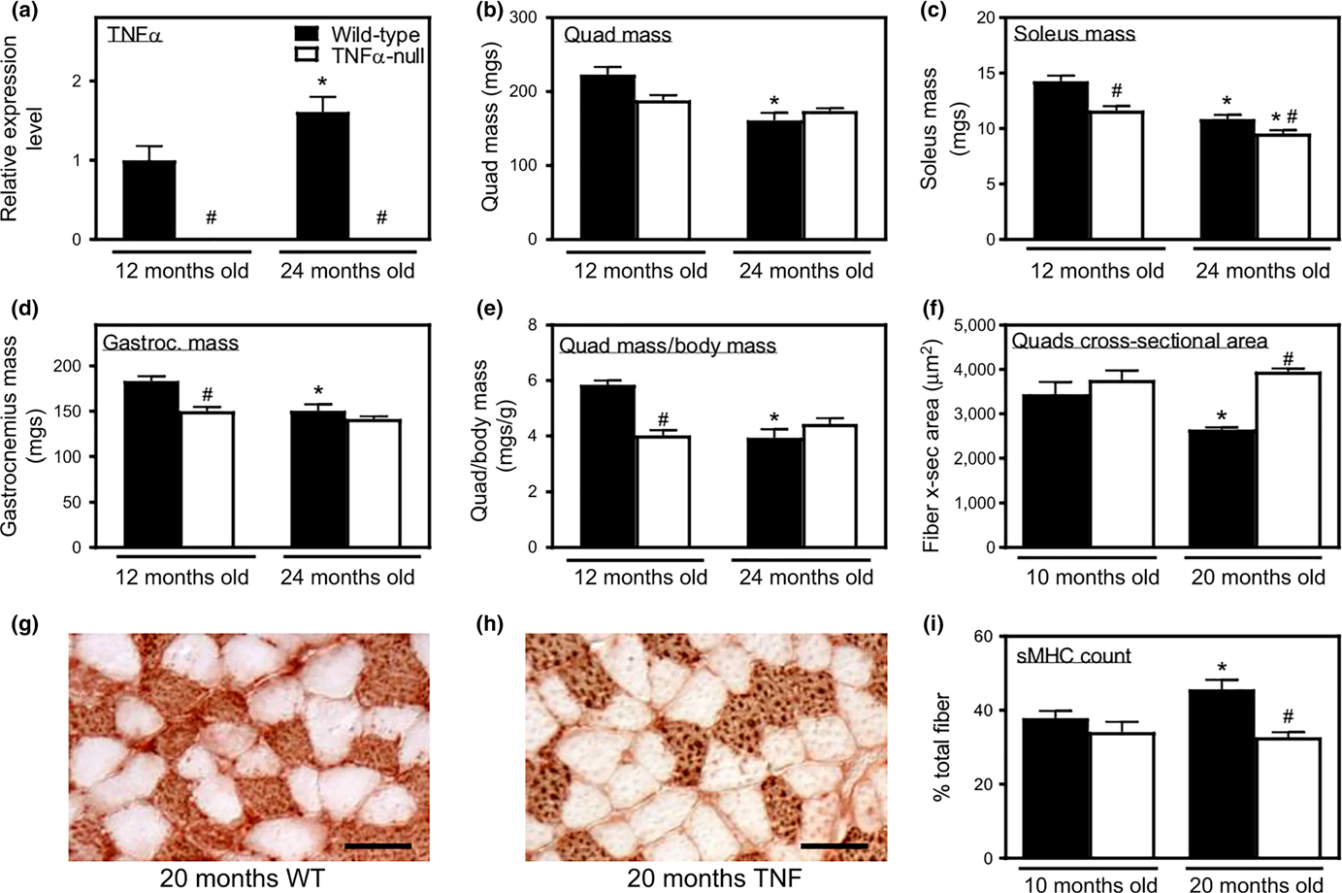
Genetic ablation of *TNF*‐α prevents sarcopenia and age‐related change in muscle fiber type composition. (a) QPCR analysis of *TNF*‐α expression in 12‐ and 24‐month‐old quadriceps muscles showed an increase in TNF‐α with aging. Values normalized to 12‐month‐old mice. (b–d) TNF‐α deficiency prevented age‐related decrease in mass of quadriceps (b), soleus (c), and gastrocnemius muscles (d). (e) Quadriceps muscle mass normalized to body mass declined from 12‐ to 24‐month‐old in wild‐type mice, but not in *TNF‐α*‐null mice. (f) *TNF‐α* mutation prevented the decrease in fiber cross‐sectional area of the quadriceps muscles in wild‐type mice from 10 to 20 months old. (g, h) Representative cross sections of soleus muscles of 20‐month‐old, wild‐type (g) and *TNF‐α‐*null (h) mice labeled with antibodies to sMHC. Bars = 50 µm. (i) The percentage of sMHC+ fibers/total fibers in soleus muscles increased from 12‐ to 24‐month‐old in wild‐type mice, but not in *TNF‐α*‐null mice. *Significant difference from young, genotype‐matched muscles at *p* < 0.05. ^#^Significant difference from age‐matched, wild‐type muscles at *p* < 0.05. *N* = 5 per data set

QPCR analysis of quadriceps muscles from adult (12 months old) and old (24 months old) wild‐type mice and showed that aging is associated with greater expression of TNF‐α (Figure 1a). We then tested whether genetic ablation of *TNF*‐*α* affected age‐related changes in muscle. *TNF*‐*α*‐null mice had no detectable expression of TNF‐α in muscles at 12 or 24 months of age (Figure 1a). Quadriceps, soleus, and gastrocnemius muscles showed significant decreases in wet muscle mass in old wild‐type mice compared to adult wild‐type mice. However, no change in muscle mass occurred during aging in *TNF*‐*α*‐null mice (Figure 1b–d). Similarly, the decrease in quadriceps mass‐to‐body mass ratio observed during aging in wild‐type mice was ablated in *TNF*‐*α‐*null animals (Figure 1e). Because reductions in muscle mass could reflect changes in the tissue other than changes in muscle fibers themselves, we also assayed for changes in the cross‐sectional area (CSA) of muscle fibers. Using this more specific assay, we found that significant sarcopenia occurred by 20 months of age (Figure 1f). Although quadriceps muscle fiber CSA decreased significantly between 10 and 20 months of age in wild‐type mice, no reduction in fiber size occurred in *TNF*‐*α* mutant mice during that period (Figure 1f). In addition, muscle fibers in 20‐month‐old, *TNF*‐*α*‐null mice were significantly larger than age‐matched wild‐type mice (Figure 1f).

Preferential atrophy and loss of fast‐twitch fibers increase the ratio of slow‐twitch fibers to total fibers in aging muscle (Deschenes, Gaertner, & O'Reilly, 2013; Larsson, Sjödin, & Karlsson, 1978). We performed slow myosin heavy chain (sMHC) staining on cross sections of soleus muscle and found that the percentage of sMHC+ fibers/total fibers increased in wild‐type mice from 10 to 20 months of age. This age‐related change of fiber composition ratio was prevented by *TNF‐α* ablation (Figure 1g,h). Together, these data show that TNF‐α contributes to muscle wasting and preferential loss of fast‐twitch fibers during aging.

### 2.2 Genetic ablation of *TNF‐α* promotes satellite cell activation in aging muscle

**Figure 2:**
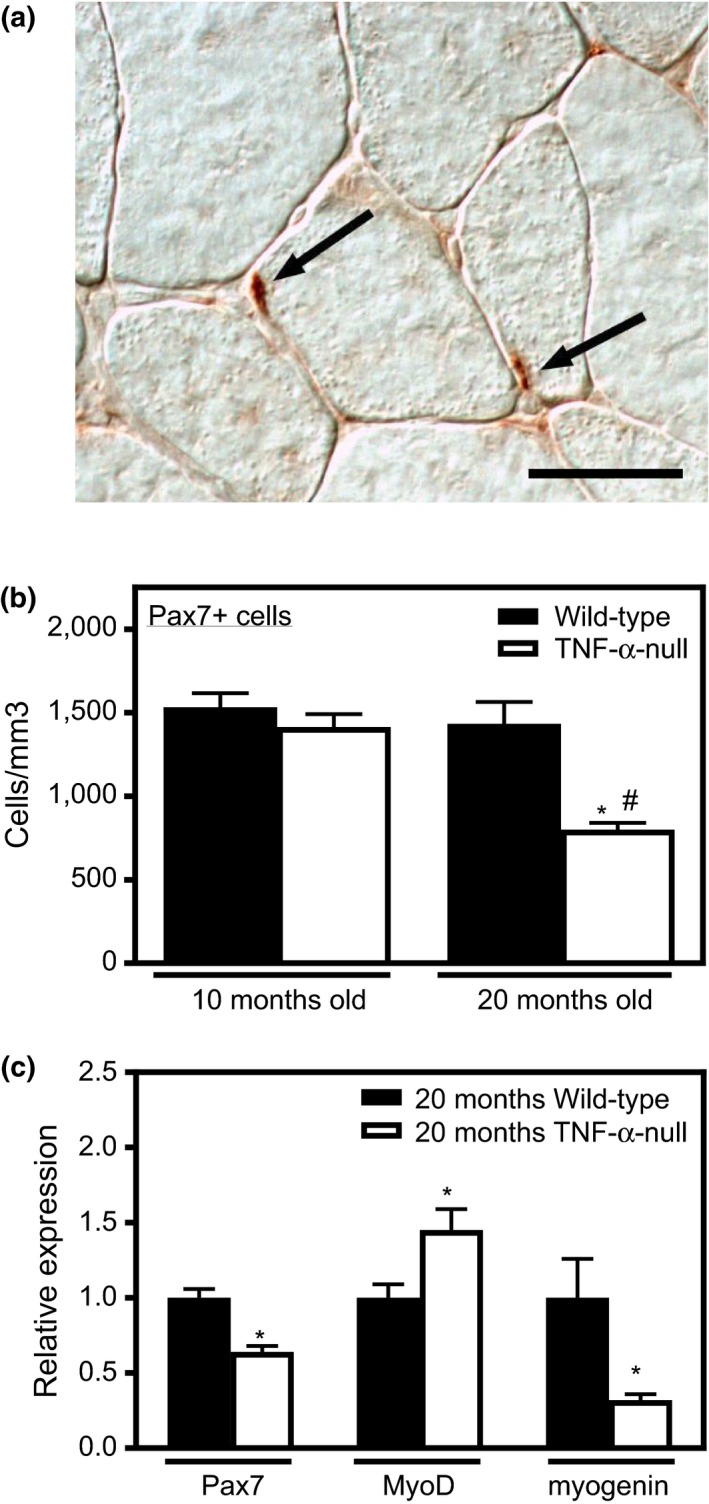
*TNF‐α* mutation promotes satellite cell activation in aging muscle in vivo. (a) Representative cross sections of quadriceps muscles of 20‐month‐old, wild‐type mice labeled with antibodies to Pax7+ satellite cells (arrows). Bar = 50 µm. (b) Aging did not affect satellite cell numbers in wild‐type mice between 10‐ and 20‐months. TNF‐α deficiency decreased satellite cell numbers at 20 months but not at 10 months. *Significant difference from 10‐month‐old, genotype‐matched muscles at *p* < 0.05. ^#^Significant difference from age‐matched, wild‐type muscles at *p* < 0.05. *N* = 5 per data set. (c) QPCR analysis of 20‐month‐old muscles in wild‐type and *TNF‐α*‐null mice showed that TNF‐α deficiency decreased Pax7 and myogenin and increased MyoD. Values normalized to 20‐month‐old wild‐type mice. *Significant difference at *p* < 0.05. *N* = 5 per data set

Because satellite cell senescence during aging can contribute to sarcopenia, we tested whether TNF‐α affects satellite cell activation in aging muscle. At 10 months of age, the number of satellite cells per unit volume (Figure 2a,b) was similar in wild‐type and *TNF*‐*α*‐null mice. However, at 20 months, *TNF‐α*‐null mice had fewer Pax7+ cells, while Pax7+ cell number in wild‐type mice remained at a similar level compared to 10‐month‐old mice (Figure 2b). Our qPCR analysis results showed that at 20 months of age, quadriceps muscle isolated from *TNF*‐*α*‐null mice had lower expression of *Pax7 *and *myogenin* and increased expression of *MyoD*, compared to muscle from wild‐type mice at the same age (Figure 2c).

### 2.3 *TNF‐α*‐deficient mice have more myonuclei in aging muscle and regenerative muscle

**Figure 3:**
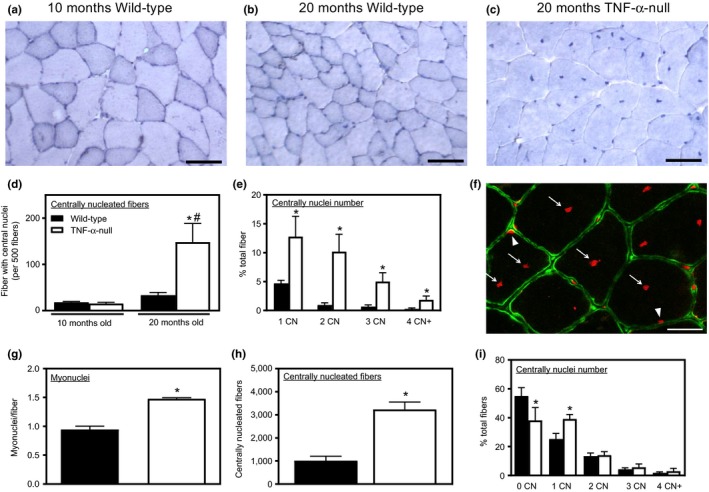
TNF‐α‐deficient mice have more nuclei in aging and regenerative muscles. (a–c) Representative images of cross sections of quadriceps muscles from 10‐month‐old, wild‐type mice (a), 20‐month‐old, wild‐type mice (b), or 20‐month‐old, *TNF*‐α‐null mice (c), showing an increased number of centrally nucleated fibers and fibers with multiple central nuclei in old *TNF*‐α‐null mice. Bars = 50 µm. (d) Old, *TNF*‐α‐null muscles have more centrally nucleated fibers. *Significant difference from 10‐month‐old, genotype‐matched muscles at *p* < 0.05. ^#^Significant difference from age‐matched, wild‐type muscles at *p < *0.05. *N* = 5 per data set. (e) Quantification of central nuclei in each fiber showed that old *TNF*‐α‐null mice have a higher percentage of fibers in each category. Bars in the column labeled “1 CN” indicate the proportion of muscle fibers with one central nucleus in total fibers measured. “2 CN” indicates the proportion of muscle fibers with two central nuclei in total fibers measured, and so forth. *Significant difference from wild‐type muscle fibers with the same number of central nuclei at *p* < 0.05. *N* = 5 per data set. (f) Immunofluorescence labeling for dystrophin (green) with propidium iodide staining (red). Arrow heads: myonuclei at the surface of a muscles fiber. Arrows: myonuclei inside a muscle fiber (central nuclei). Bar = 25 µm. (g) Quantification of total myonuclei in each fiber showed that old *TNF‐α*‐null mice have more myonuclei per fiber compared to age‐matched wild‐type mice. *Significant difference at *p* < 0.05. (h) *TNF*‐α ablation increased the number of centrally nucleated fibers at 21 days after injury. *N* = 5 per data set. *Significant difference at *p* < 0.05. (i) Total fiber numbers within the injury site were counted in wild‐type and *TNF*‐α‐null muscles at 21 days after injury. Muscle fibers with 0, 1, 2, 3, and 4 or more central nuclei were quantified and expressed as proportion of total fibers. *Significant difference from wild‐type muscle fibers with the same number of central nuclei at *p* < 0.05. *N* = 5 per data set

Activated satellite cells proliferate, differentiate, and then fuse with existing muscle fibers to contribute to myogenesis following muscle injury, creating centrally nucleated fibers that provide an index of muscle regeneration. The number of centrally nucleated fibers was very low in healthy noninjured quadriceps muscle of both adult and old wild‐type mice (Figure 3a,b,d). However, more than 30% of the muscle fibers in old, *TNF*‐*α*‐null mice quadriceps muscles were centrally nucleated fibers (Figure 3c,d). Furthermore, many of the centrally nucleated fibers showed more than one central nucleus in a single plane of section, indicating an abnormal, hypernucleated state (Figure 3c). The percentages of fibers with 1, 2, 3, or 4 and more central nuclei increased significantly in old *TNF*‐*α*‐null mice compared to old wild‐type mice (Figure 3e). Because the observed increase in central nuclei in old *TNF*‐*α*‐null muscles could be caused by increased muscle cell fusion and by translocation of myonuclei from their normal location near the fiber surface to a central location, we measured total number of myonuclei in muscle cross sections, including both central and peripheral nuclei in the counts (Figure 3f,g). Total number of myonuclei per muscle fiber in cross sections increased in *TNF*‐*α*‐null mice, which validated an increase in fusion caused by the *TNF*‐*α* mutation (Figure 3g). These results suggested that TNF‐α deficiency increases muscle cell fusion with muscle fibers, leading to more central‐nucleated fibers as well as increased nuclei per fiber.

We then performed acute injury to muscles of healthy, adult *TNF*‐*α*‐null mice to test whether the increase in central nucleation was restricted to aging muscle. Quadriceps muscles of 12‐month‐old wild‐type and *TNF*‐*α*‐null mice were injured by BaCl_2_ injection and central nucleation was quantified 21 days after injury. Our results showed that injured muscles from *TNF*‐*α*‐null mice had more centrally nucleated fibers compared to wild‐type muscles (Figure 3h,i).

### 2.4 Myoblasts isolated from *TNF‐α*‐null mice are more fusogenic than wild‐type myoblasts

**Figure 4:**
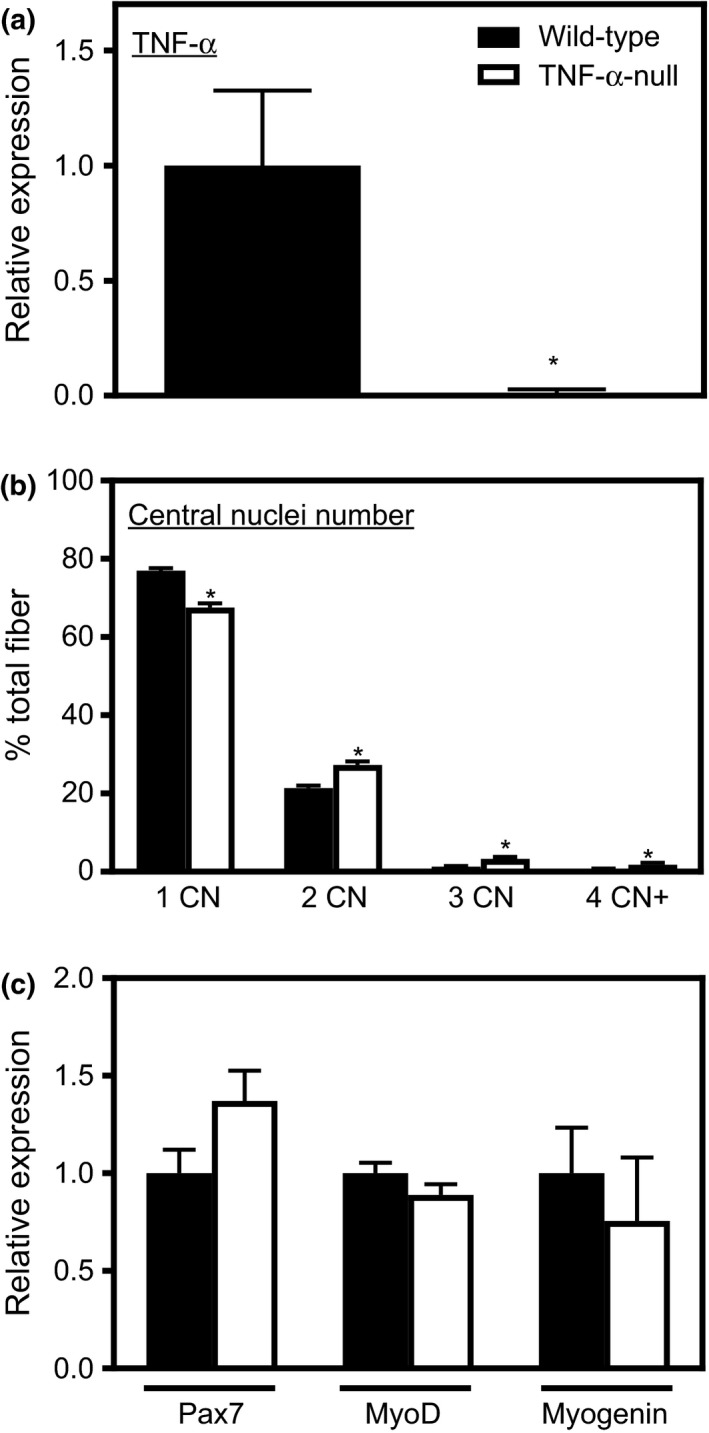
*TNF*‐α ablation promoted myoblast fusion*.* (a) QPCR analysis showed no detectable *TNF*‐α expression in primary myoblasts isolated from *TNF‐α*‐null mice. *Significant difference from wild‐type myoblasts at *p* < 0.05. *N* = 6 per data set. (b) *TNF*‐α‐null myotubes have more nuclei compared to wild‐type. *Significant difference from wild‐type myotubes at *p* < 0.05. Results are from three independent experiments. *N* = 6 per data set. (c) QPCR analysis of primary myoblasts isolated from wild‐type and *TNF‐α‐*null mice showed no difference in Pax7, MyoD, or myogenin mRNA. Values normalized to myoblasts isolated from wild‐type mice. *N* = 5 per data set

We tested whether muscle‐derived TNF‐α inhibited satellite cell fusion by analyzing in vitro primary cell cultures. First, we validated that wild‐type myoblasts expressed TNF‐α in vitro (Figure 4a). We then found that myoblasts isolated from *TNF*‐*α‐*null mice started fusing and forming myotubes more rapidly than myoblasts from wild‐type mice and at Day 6 after induction of differentiation myotubes formed by myoblasts from *TNF*‐*α*‐null mice had more nuclei (Figure 4b). These data support our in vivo observation that TNF‐α deficiency promotes muscle cell fusion and increases myonuclei numbers. However, our qPCR analysis of primary muscle cells showed no significant differences in Pax7, MyoD, or myogenin expression between wild‐type and *TNF*‐*α*‐null mice (Figure 4c). Together, these findings indicate that TNF‐α deficiency in satellite cells contributes directly to their increased fusion capacity, without affecting their expression of key transcription factors that influence muscle differentiation.

### 2.5 TNF‐α secreted by macrophages affects the fusion capacity of muscle cells

**Figure 5:**
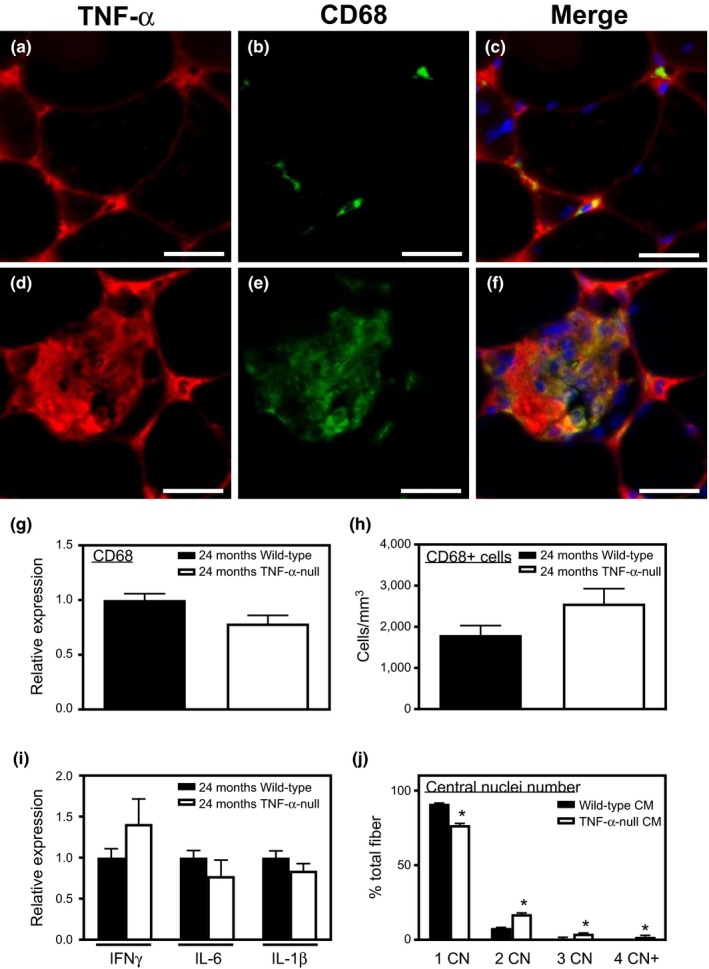
TNF‐α secreted by macrophages affects the fusion capacity of muscle cells. (a–f) Immunofluorescent, double‐labeling for TNF‐α and CD68. Sections of 20‐month‐old wild‐type quadriceps muscle labeled with anti‐TNF‐α (red; panel a, d) and anti‐CD68 (green; panel b, e) and the merged images (yellow; panel c, f) showed that CD68+ cells expressed TNF‐α. Nuclei are stained blue with DAPI. Bars = 20 µm. (g) QPCR analysis showed no difference in CD68 mRNA expression between 24‐month‐old wild‐type and *TNF*‐α‐null quadriceps muscles. *N* = 5 per data set. (h) Counts for CD68+ cells showed that *TNF*‐α ablation did not affect macrophage numbers in aging muscle. *N* = 5 per data set. (i) QPCR analysis showed that *TNF*‐α ablation did not affect the expression of IFNγ, IL‐6, or IL‐1β. (j) Quantification of nuclei per myotube showed that cells receiving conditioned media from *TNF*‐α‐null BMDM had more nuclei. Wild‐type CM: media conditioned by wild‐type BMDMs. *TNF*‐α‐null CM: media conditioned by *TNF*‐α‐null BMDMs. *Significant difference from wild‐type myofibers with the same number of nuclei at *p* < 0.05. *N* = 6 per data set

Our previous investigations showed that macrophage numbers increase in aging muscle, consistent with a systemic increase in proinflammatory cytokines during aging (Wang et al., 2015). We assayed for TNF‐α expression in old muscle by immunofluorescence and found that CD68+ macrophages in the muscle of healthy old mice express TNF‐α (Figure 5a–c). We also observed that TNF‐α accumulated in the connective tissue surrounding muscle fibers. Moreover, necrotic fibers that were infiltrated by CD68+ macrophages also showed high expression of TNF‐α (Figure 5d–f). Unsurprisingly, TNF‐α was not detectable in TNF‐α‐null muscles (data not shown). There was no difference in either CD68 mRNA expression or the number of CD68+ macrophages in old wild‐type and *TNF*‐*α*‐null mice (Figure 5g,h). We also tested whether *TNF‐α* ablation affected the expression of other inflammatory cytokines that can affect myogenesis (Authier et al., 1999; Lieskovska, Guo, & Derman, 2003). Our qPCR results showed that the expression of IFNγ, IL‐6, and IL‐1β did not differ significantly in 24‐month‐old, *TNF*‐*α‐*null muscles compared to age‐matched wild‐type muscles (Figure 5i). Thus, *TNF*‐*α*‐null mice have normal expression of other proinflammatory cytokines that can influence myogenesis, despite the deficiency of TNF‐α*. *The results also show that changes in muscle fiber size and satellite cell numbers during aging that were caused by *TNF*‐*α *ablation were not attributable to changes in macrophage numbers or levels of other inflammatory cytokines. We next tested whether TNF‐α deficiency in macrophages can contribute to increased activation and fusion of muscle cells. Conditioned media were collected from bone marrow‐derived macrophage (BMDM) cultures from adult wild‐type and *TNF*‐*α*‐null mice. Muscle cells were treated with conditioned media for 2 days before induction of differentiation, followed by another 5 days of treatment with conditioned media after induction of differentiation. Muscle cells receiving conditioned media from BMDMs isolated from *TNF*‐*α*‐null mice had more nuclei per fiber than cultures receiving media conditioned by wild‐type BMDMs (Figure 5j). This shows that macrophage production of TNF‐α influences muscle cell fusion although changes in the expression of other, untested proteins that occurred as a result of *TNF*‐*α* deletion could also be involved in the treatment effects we observed.

### 2.6 Transplantation of wild‐type BMCs into *TNF‐α*‐null mice reduced myonuclei numbers and increased sarcopenia

**Figure 6:**
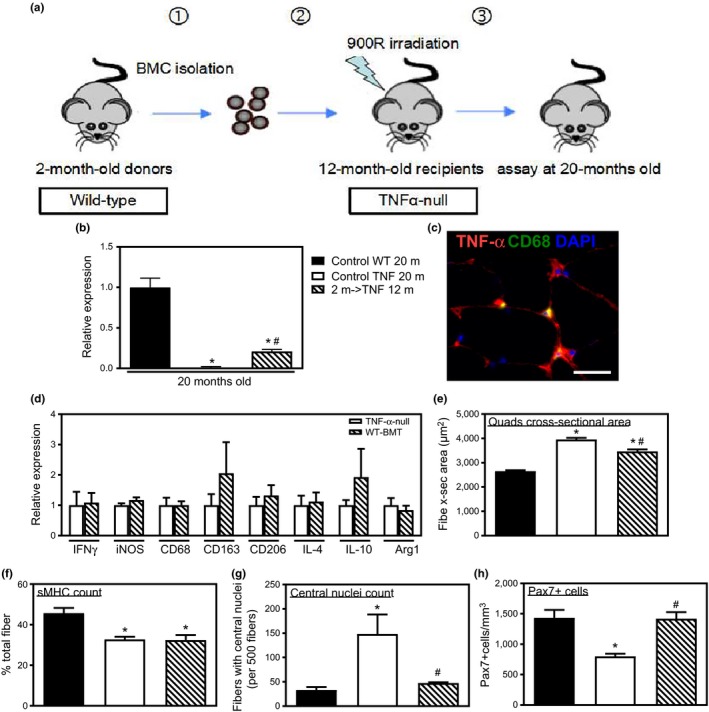
Effects of BMT of wild‐type BMCs into *TNF*‐α‐null mice on myonuclei numbers and muscle aging. (a) Schematic of experimental design for BMT experiments. (b) QPCR analysis of *TNF*‐α expression in quadriceps muscle from 20‐month‐old wild‐type mice, *TNF*‐α‐null mice, and *TNF*‐α ‐null mice receiving BMT. Wild‐type BMT induced TNF‐α expression in *TNF*‐α‐null mice. Values normalized to 20‐month‐old wild‐type mice. (c) Cross section of quadriceps muscle of 20‐month‐old *TNF*‐α‐null mice that received wild‐type BMT when the recipients were 12 months of age. Section labeled with anti‐TNF‐α (red) and anti‐CD68 (green) showed that BMT recipients had detectable TNF‐α at the muscle fiber surface (red) and within macrophages (yellow). Bar = 25 µm. (d) QPCR analysis of IFNγ, IL‐4, IL‐10, iNOS, CD68, CD163, CD206, and Arg1 showed that wild‐type BMT into *TNF*‐α‐null did not affect expression of cytokines and macrophage‐related genes compared to nontransplanted, *TNF*‐α‐null mice. (e) Increased muscle fiber CSA in 20‐month‐old *TNF*‐α‐null compared to age‐matched, wild‐type mice was reduced by wild‐type BMT. (f) 20‐month‐old *TNF*‐α‐null mice had lower percentage of sMHC+ muscle fibers in soleus muscles compared to 20‐month‐old wild‐type mice; the decrease was not rescued by wild‐type BMT. (g) Centrally nucleated fibers increased in number in 20‐month‐old *TNF*‐α‐null mice compared to wild‐type mice, but the increase did not occur in 20‐month‐old *TNF*‐α‐null mice that had received wild‐type BMT when the recipients were 12 months of age. (h) Anti‐Pax7 immunohistochemistry showed that satellite cell number decreased in 20‐month‐old *TNF*‐α‐null mice compared to wild‐type mice. The increase was ablated by wild‐type BMT. For all panels, *Significant difference from 20‐month‐old wild‐type muscles at *p* < 0.05. ^#^Significant difference from 20‐month‐old *TNF*‐α‐null muscles at *p* < 0.05. *N* = 5 per data set

Because macrophages from *TNF*‐*α*‐null mice increased myoblast fusion, we tested whether TNF‐α secreted by myeloid cells is important in muscle aging by cross‐genotype bone marrow transplantation (BMT; Figure 6a). QPCR analysis and immunofluorescence showed that *TNF*‐*α*‐null mice that received wild‐type BMCs had elevated expression of TNF‐σ in skeletal muscle (Figure 6b,c). Wild‐type BMT into *TNF*‐*α*‐null mice did not affect mRNA expression of pro‐ and anti‐inflammatory cytokines or macrophage‐related genes (Figure 6d).

Twenty‐month‐old *TNF*‐*α*‐null mice that received wild‐type BMT showed a partial restoration of sarcopenia. Although *TNF*‐*α* ablation caused 49% larger CSA of quadriceps muscle fibers in 20‐month‐old mice compared to wild‐type mice, BMT of TNF‐α‐expressing BMCs into *TNF*‐*α*‐null recipients produced fiber CSAs that were only 33% larger than controls (Figure 6e). However, the decrease in sMHC+ fiber percentage in soleus muscles induced by TNF‐α deficiency was not prevented by wild‐type BMT (Figure 6f). In addition, wild‐type BMT into old *TNF*‐*α‐*null mice reduced the number of centrally nucleated fibers compared to old, nontransplanted, *TNF*‐*α*‐null mice (Figure 6g). We also found that wild‐type BMT into old *TNF*‐*α*‐null mice prevented the reduction in satellite cell numbers observed in nontransplanted, old *TNF*‐*α*‐null mice compared to old wild‐type mice (Figure 6h). In summary, these results indicate that BMCs from wild‐type mice maintained satellite cell numbers and reduced cell fusion while increasing sarcopenia.

## 3 DISCUSSION

Relationships between elevations in serum TNF‐α in chronic diseases and the occurrence of more rapid muscle wasting have been known for many years (Beutler & Cerami, 1986). Similarly, associations between elevated levels of circulating, proinflammatory cytokines and the loss of muscle mass and function during aging are well‐established (Visser et al., 2002). However, whether changes in circulating, proinflammatory cytokines cause or merely correlate with muscle wasting during aging is not established. The primary, novel finding of the present investigation is that TNF‐α expression in normal, healthy, aging animals plays a significant role in influencing sarcopenia and satellite cell numbers. In the systemic absence of TNF‐α, sarcopenia is slowed and satellite cell numbers decline. Furthermore, our findings show that TNF‐α expressed by myeloid cells contributes significantly to sarcopenia and is sufficient to maintain satellite cell numbers in aging, *TNF‐α‐*null mice. Thus, the systemic shift of the innate immune system to a more proinflammatory status that occurs during inflammaging may cause loss of muscle mass and satellite cell numbers through mechanisms driven by TNF‐α produced by myeloid cells.

The finding that the systemic ablation of *TNF‐α* resulted in more central nuclei and total nuclei in aging muscle fibers was particularly intriguing to us. Adult skeletal muscle is a fully differentiated tissue with little turnover of nuclei and central nucleation of muscle fibers typically occurs only in muscle fibers that have regenerated after injury or disease (Charge & Rudnicki, 2004). Our observation that over 30% of myofibers in noninjured quadriceps muscles of *TNF‐α‐*null mice were central‐nucleated suggests that in a TNF‐α‐deficient environment, satellite cells undergo increased fusion with existing myofibers without exogenous stimuli such as muscle injury. This interpretation is supported by our in vitro findings that *TNF*‐*α*‐null muscle cells show greater numbers of nuclei per cell than wild‐type muscle cells. Furthermore, 20‐month‐old *TNF*‐*α*‐null mice showed 45% fewer Pax7+ satellite cells in comparison with age‐matched, wild‐type mice, which may also reflect a more avid fusion with muscle fibers in the absence of TNF‐α.

The increased nucleation of muscle fibers in old, *TNF*‐*α*‐null mice and the slowing of sarcopenia suggest that the two outcomes may be mechanistically related, although our in vivo data cannot definitively address the question. However, linkage of myonuclei number and muscle fiber size is the basis of the “myonuclear domain” hypothesis which asserts that a single myonucleus controls the translational and transcriptional regulation of protein synthesis for a limited cell volume known as the myonuclear domain. Although more recent studies indicate that age‐related myofiber atrophy results primarily from reductions in myonuclear domain size instead of the loss of myonuclei numbers (Karlsen et al., 2015; Schwartz, Brown, McLaughlin, Smith, & Bigelow, 2016), other findings show that increased myonuclear fusion facilitates muscle hypertrophy, especially when the myonuclear domain size exceeds a certain threshold (Jo et al., 2012; Petrella, Kim, Cross, Kosek, & Bamman, 2006). The findings in the current study support the possibility that the ablation of *TNF‐α *contributes to increased myofiber size in aging muscle by increasing the frequency of muscle cell fusion with aging muscle fibers.

Our finding that slowing sarcopenia in *TNF*‐*α*‐null mice occurred while there was an accelerated loss of satellite cells also relates to our developing understanding of the relationships between satellite cell numbers and the regulation of muscle mass during aging. Although the age‐related decline in satellite cell number contributes to the reduced regenerative capacity of injured, aging muscle (Brack, Bildsoe, & Hughes, 2005; Fry et al., 2015), whether satellite cell loss is sufficient to cause reduction in muscle mass in uninjured, aging muscle is controversial. Targeted ablation of Pax7+ satellite cells that produced a lifelong reduction in satellite cell numbers by approximately 70%–90% did not increase sarcopenia in sedentary mice (Fry et al., 2015) and findings in the current study show that satellite cell numbers can be reduced by about 45% in aging muscle, which leads to a reduction in sarcopenia. However, neither investigation completely eliminated satellite cells. In addition, genetic ablation of Pax7‐expressing cells caused deletion of satellite cells early in their myogenic lineage (Fry et al., 2015), while the current investigation caused significant reductions in satellite cell numbers only in older mice, apparently by biasing them toward a more differentiated state. Thus, the relationship between satellite cell numbers and sarcopenia may be influenced by the presence of injury, the magnitude and age at which satellite cell numbers are reduced and by the method used for their reduction.

Although the mechanisms through which *TNF‐α* ablation contributes to loss of satellite cell numbers in vivo are unknown, we hypothesize that the bimodal role of TNF‐α in regulating myogenesis may underlie this effect. TNF‐α promotes myoblast proliferation at early stages of myogenesis while repressing myoblast differentiation (Chen et al., 2007; Guttridge et al., 2000; Langen et al., 2001; Li, 2003). Administration of TNF‐α to primary myoblast cultures increased number of primary myoblasts incorporating BrdU (Li, 2003) and increased myoblast proliferation in C2C12 cultures (Alvarez et al., 2002). However, administration of TNF‐α to myoblast cultures after induction of differentiation inhibited the formation of myotubes, decreased the total protein content, and decreased differentiation (Langen et al., 2001). Thus, loss of *TNF*‐*α *expression in aging muscle in vivo could reduce the proliferation of satellite cells and promote their differentiation and fusion, which could produce the phenotype we observe in old, *TNF*‐*α *mutant muscles.

The effects that we observe in aging muscle where there has been a systemic ablation of *TNF*‐*α *or where TNF‐α has been partially restored through BMT of wild‐type BMCs are consistent with TNF‐α ‐mediated effects on satellite cell proliferation and differentiation. However, manipulation of TNF‐α levels either by *TNF*‐*α* mutation or by BMT of TNF‐α‐expressing cells will likely have other effects on muscle fibers that would also influence the phenotype that we observe. For example, TNF‐α affects muscle mass through its catabolic role in regulating muscle protein content. TNF‐α treatment of differentiated myotubes activates NFκB, which can lead to reductions in protein content (Li, Schwartz, Waddell, Holloway, & Reid, 1998). TNF‐α also induces the ubiquitin‐proteasome system in cachexia, which can be attenuated by blocking the activation of NFκB signaling (Reid & Li, 2001). TNF‐α can also decrease muscle protein content by inhibiting protein synthesis through the induction of IL‐6 or inhibition of insulin‐like growth factor‐I signaling (Alvarez et al., 2002; Frost, Lang, & Gelato, 1997). Increased TNF‐α levels can induce apoptosis in disease models and during muscle aging through the increase in cell death‐inducing receptor, Fas (CD95), and the interaction of the TNF‐α receptor complex and the Fas‐associated protein with death domain (Lees, Zwetsloot, & Booth, 2009; Li et al., 1998). Thus, multiple mechanisms may underlie the reduction in sarcopenia in TNF‐α‐mutant mice, in addition to the effects we report concerning influences on satellite cell function and muscle cell fusion during aging.

Another intriguing finding in the present study is that we showed that TNF‐α secreted by both satellite cells and macrophages plays important roles in regulating myogenesis. An increased fusion index and ability to form myotubes in vitro was seen in both primary myoblast isolated from *TNF‐α‐*null mice and C2C12 myoblast cells cultured with conditioned media from BMDMs isolated from *TNF*‐*α*‐null mice. These data suggest that TNF‐α can regulate satellite cell function through both autocrine and paracrine regulation. Moreover, we showed that *TNF*‐*α*‐null mice that received BMT of wild‐type BMCs exhibited smaller muscle fiber size at 20 months of age compared to *TNF*‐*α*‐null mice without BMT. Transplantation of wild‐type BMCs into *TNF*‐*α*‐null mice also restored the number of satellite cells in aging muscle and inhibited the hyperfusion of muscle cells seen in nontransplanted *TNF*‐*α*‐null mice. These results indicate that myeloid cell‐derived TNF‐α contributes to muscle aging by regulating satellite cell function. However, transplantation of wild‐type BMCs into *TNF*‐*α*‐null mice was insufficient to restore the age‐related changes in fiber type, showing that TNF‐α derived from other cell types, like aging muscle cells, also contributes to muscle aging.

Our study indicates a novel strategy for reducing age‐related changes in muscle, especially sarcopenia, by manipulating the immune system. Experimental or therapeutic modulation of TNF‐α, which plays an important role in regulating satellite cell fusion in aging muscle, may provide a particularly useful target for reducing age‐related changes in muscle. Future investigations are needed to fully elaborate the mechanism through which TNF‐α contributes to muscle aging and the translational potential of reducing sarcopenia through TNF‐α‐related therapies.

## 4 EXPERIMENTAL PROCEDURES

A more detailed account of experimental procedures can be found in the online Supporting Information Appendix S1 accompanying this article.

### 4.1 Animal treatments

Experiments involving mice were conducted according to the National Institutes of Health (NIH) Guide for the Care and Use of Laboratory Animals and were approved by the University of California, Los Angeles Institutional Animal Care and Use Committee. Following euthanasia, muscles were collected from wild‐type mice (C57 BL/6) and *TNF*‐α‐null mice, weighed and then flash‐frozen for subsequent sectioning and histological evaluation or used for RNA isolation. Other mice were used for BMT in which donor mice were 2‐month‐old, female mice, and recipient mice were 12‐month‐old male mice. Recipient mice were subjected to myeloablative irradiation prior to BMT. Muscles and blood were collected from recipient mice 8 months following BMT.

### 4.2 Histology and immunohistochemistry

Frozen cross sections were cut from the midbelly of quadriceps muscles at a thickness of 10 μm. Hematoxylin‐stained sections were used to quantify muscle fiber CSA and central nucleation. Total nucleation was quantified in sections immunolabeled with antidystrophin and propidium iodide staining, as described in Supporting Information Appendix S1.

Other frozen sections were immunolabeled with rabbit anti‐sMHC, rat anti‐CD68, or mouse anti‐Pax7 and then used to quantify numbers of slow muscle fibers, macrophages, or satellite cells, respectively. Sections were also double‐labeled with anti‐CD68 and mouse anti‐TNF‐α to identify TNF‐α‐expressing macrophages, as described in Supporting Information Appendix S1.

### 4.3 RNA isolation and quantitative PCR

Frozen muscles were homogenized and RNA was isolated, cDNA generated, and QPCR performed as described previously (Villalta et al., 2011). Primers used for QPCR are listed in Supporting Information Table S1.

### 4.4 Primary myoblast isolation and fusion assay

Primary myoblasts were isolated following a previous protocol (Wehling‐Henricks et al., 2016) and detailed in Supporting Information Appendix S1 and then cultured on coverslips. After 6 days of growth in differentiation medium to induce cell fusion, cells on the coverslips were immunolabeled with rabbit antidesmin. The number of myonuclei per myotube was counted for 500 myotubes sampled randomly on the coverslips, to determine a fusion index.

### 4.5 Muscle cell fusion assay with conditioned media from BMDMs

Bone marrow cells were isolated following a previously described protocol (Wang et al., 2015; detailed in Supporting Information Appendix S1) and differentiated to form BMDMs. The BMDMs were then stimulated for 24 hr with activation media containing 10 ng/ml MCSF. Conditioned media were collected following activation and added to myoblast cultures. Two days after culture in conditioned media, cells were cultured in DMEM only overnight followed by culture in BMDM‐conditioned media for 5 days, with media changed every 36 hr. Coverslips were then collected for desmin staining and fusion index quantification.

### 4.6 Statistics

Data are presented as mean ± *SEM*. One‐way analysis of variance was used to test whether differences between three or more groups were significant at *p* < 0.05. Significant differences were identified using Tukey's post hoc test. Comparisons of two groups of values were analyzed using the unpaired, two‐tailed *t* test.

## Conflict of Interest

None declared.

## AUTHOR CONTRIBUTIONS

All authors participated in experimental design, experimentation, data analysis, and preparation of the manuscript.
