# Supplementary figures and images for: Myeloid cell‐derived tumor necrosis factor‐alpha promotes sarcopenia and regulates muscle cell fusion with aging muscle fibers
Source: Aging Cell. 2018 Sep 6;17(6):e12828. doi: 10.1111/acel.12828 (PMC6260911; doi:10.1111/acel.12828)

Supplemental figure.

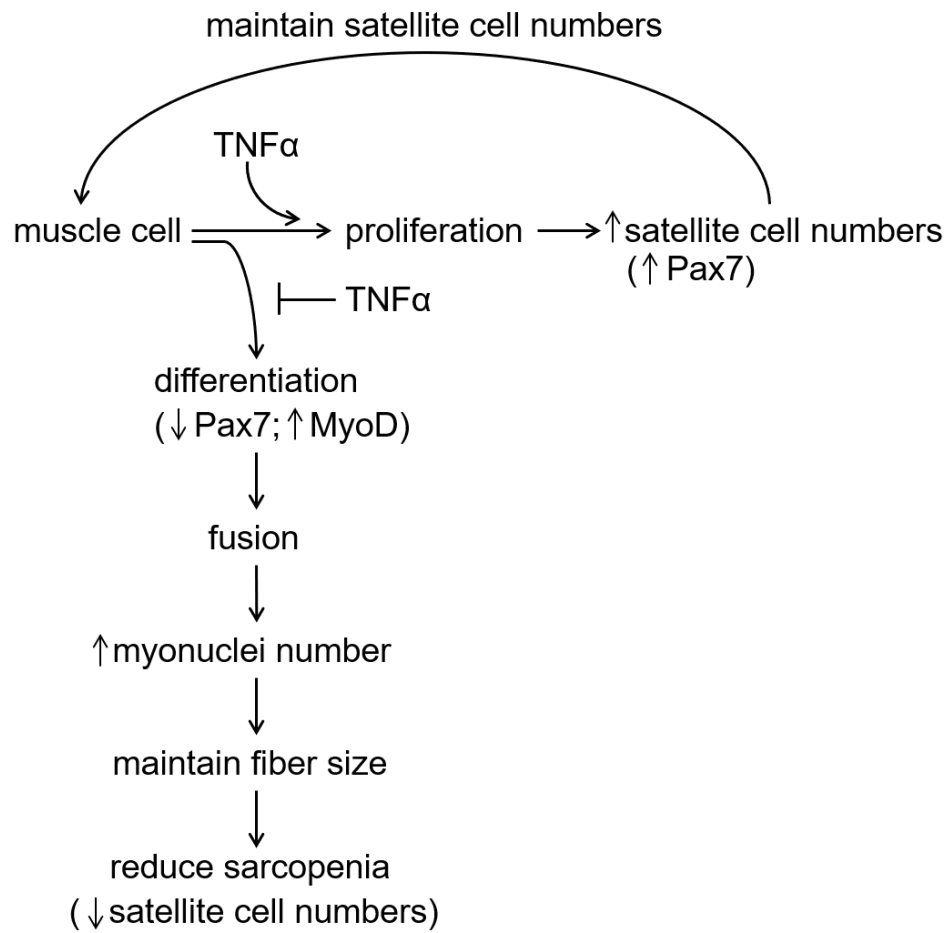

Supplement: Supplementary file 1 [file ACEL-17-e12828-s001.pdf]
