# Supplementary material for: Myeloid cell‐derived tumor necrosis factor‐alpha promotes sarcopenia and regulates muscle cell fusion with aging muscle fibers
Source: Aging Cell. 2018 Sep 6;17(6):e12828. doi: 10.1111/acel.12828 (PMC6260911; doi:10.1111/acel.12828)
Supplement: Supplementary file 2 [file ACEL-17-e12828-s002.docx]

# Supplementary Table 1 QPCR primers

| Gene | Accession Number |  | Direction (5’->3’) |
| --- | --- | --- | --- |
| Arg1 | NM_007482 | Fwd | CAATGAAGAGCTGGCTGGTGT |
|  |  | Rev | GTGTGAGCATCCACCCAAATG |
| CD163 | NM_053094.2 | Fwd | GCAAAAACTGGCAGTGGG |
|  |  | Rev | GTCAAAATCACAGACGGAGC |
| CD206 | NM_002438 | Fwd | GGATTGTGGAGCAGATGGAAG |
|  |  | Rev | CTTGAATGGAAATGCACAGAC |
| CD68 | NM_001291058.1 | Fwd | CAAAGCTTCTGCTGTGGAAAT |
|  |  | Rev | GACTGGTCACGGTTGCAAG |
| IFNγ | NM_008337.3 | Fwd | GACAATCAGGCCATCAGCAAC |
|  |  | Rev | CGGATGAGCTCATTGAATGCTT |
| IL-1β | NM_008361.4 | Fwd | GTAATGAAAGACGGCACACC |
|  |  | Rev | CTCTGCAGACTCAAACTCC |
| IL-4 | NM_021283.2 | Fwd | GGATGTGCCAAACGTCCTC |
|  |  | Rev | GAGTTCTTCTTCAAGCATGGAG |
| IL-6 | NM_031168.1 | Fwd | GAACAACGATGATGCACTTGC |
|  |  | Rev | CTTCATGTACTCCAGGTAGCTATGGT |
| IL-10 | NM_010548.2 | Fwd | CAAGGAGCATTTGAATTCCC |
|  |  | Rev | GGCCTTGTAGACACCTTGGTC |
| iNOS | NM_010927.3 | Fwd | CAGCACAGGAAATGTTTCAGC |
|  |  | Rev | TAGCCAGCGTACCGGATGA |
| MyoD | NM_010866.2 | Fwd | GAGCGCATCTCCACAGACAG |
|  |  | Rev | AAATCGCATTGGGGTTTGAG |
| Myogenin | NM_031189.2 | Fwd | CCAGTACATTGAGCGCTAC |
|  |  | Rev | ACCGAACTCCAGTGCATTGC |
| Pax7 | NM_011039.2 | Fwd | CTCAGTGAGTTCGATTAGCCG |
|  |  | Rev | AGACGGTTCCCTTTGTCGC |
| PPIA | [NM_00](https://www.ncbi.nlm.nih.gov/entrez/viewer.fcgi?db=nucleotide&id=999844030)8907.1 | Fwd | GCAAATGCTGGACCAAACAC |
|  |  | Rev | TCACCTTCCCAAAGACCACAT |
| RNPS1 | NM_001080127.1 | Fwd | AGGCTCACCAGGAATGTGAC |
|  |  | Rev | CTTGGCCATCAATTTGTCCT |
| SRP14 | NM_009273.4 | Fwd | AGAGGCGAGCAGTTCCTGAC |
|  |  | Rev | CGGTGCTGATCTTCCTTTTC |
| TGFβ | NM_011577.1 | Fwd | CTCCACCTGCAAGACCAT |
|  |  | Rev | CTTAGTTTGGACAGGATCTGG |
| TNFα | NM_013693.3 | Fwd | CTTCTGTCTACTGAACTTCGGG |
|  |  | Rev | CACTTGGTGGTTTGCTACGAC |
| TPT1 | NM_009429.3 | Fwd | GGAGGGCAAGATGGTCAGTAG |
|  |  | Rev | CGGTGACTACTGTGCTTTCG |
