# Supplementary material for: Myeloid cell‐derived tumor necrosis factor‐alpha promotes sarcopenia and regulates muscle cell fusion with aging muscle fibers
Source: Aging Cell. 2018 Sep 6;17(6):e12828. doi: 10.1111/acel.12828 (PMC6260911; doi:10.1111/acel.12828)
Supplement: Supplementary file 3 [file ACEL-17-e12828-s003.docx]

Supplemental experimental procedures.

*Mice*

All experiments involving mice were conducted according to the National Institutes of Health Guide for the Care and Use of Laboratory Animals and were approved by the University of California, Los Angeles Institutional Animal Care and Use Committee. Wild-type (WT) mice (C57 BL/6) and *TNFα*-null mice (B6.129S-*Tnf ^tm1Gkl^*/J) were obtained from Jackson Laboratories (Bar Harbor, ME, USA) or from the National Institute on Aging mouse colony.

*Dissection and muscle mass measurement*

WT and *TNFα*-null mice were euthanized by inhalation of isoflurane. Body weights were measured immediately after euthanization to an accuracy of 0.1 g. The hind-limb muscles were dissected and wet weight was determined to an accuracy of 1 mg. Muscles were then either frozen in liquid nitrogen or embedded in optimal cutting temperature compound and rapidly frozen in isopentane cooled in liquid nitrogen.

*Cross-sectional area measurement*

Frozen cross-sections were cut from the mid-belly of quadriceps femoris muscle at a thickness of 10 μm. Sections were then stained with hematoxylin (Vector) for 10 minutes. The muscle fiber cross-sectional area was measured for 500 fibers randomly sampled from complete cross-sections using a digital imaging system (Bioquant).

*Muscle fiber central nuclei counting*

Hematoxylin staining was performed on quadriceps muscle sections. The number of central nuclei was counted for each fiber for a total of 500 fibers that were randomly sampled throughout the entire cross-section.

*Myonuclei counting*

Frozen cross-sections were cut from the mid-belly of quadriceps or soleus muscle at a thickness of 10 μm. The frozen sections were air-dried for 30 minutes and fixed in ice-cold acetone for 10 minutes. Sections were then blocked with a mouse-on-mouse immunohistochemistry kit (Vector) and then immunolabeled with mouse-anti-dystrophin (Novacastra) and with propidium iodide (1:50, Trevigen) for 3 hours at room temperature. Sections were washed with phosphate buffered saline solution (PBS) and then stained with Dylight 400-anti-mouse IgG (1/200; Vector) for 30 minutes, followed by PBS wash. Myonuclei were characterized as propidium iodide positive nuclei within the anti-dystrophin-stained muscle fiber. The number of myonuclei were counted for each fiber for a total of 500 fibers that were randomly sampled throughout the entire cross-section.

*sMHC and CD68 immunohistochemistry and counting*

Acetone-fixed, frozen sections were treated with 0.3% H_2_O_2_ to quench endogenous peroxidase activity. Sections were then blocked in 3% BSA and 2% gelatin in 50 mM Tris buffer (pH 7.2) for 1 hour and then immunolabeled with rabbit anti-slow myosin heavy chain (Abcam) or rat anti-CD68 (Serotec), for 3 hours at room temperature. Sections were washed with PBS and then probed with biotin-conjugated secondary antibodies (1/200; Vector) for 30 minutes. Sections were subsequently washed with PBS and then incubated for 30 minutes with avidin D-conjugated horseradish peroxidase (1/1000; Vector). Staining was visualized with the peroxidase substrate 3-amino-9-ethylcarbazole (AEC kit; Vector), yielding a red reaction product. For fiber type composition assay, images were taken from sMHC stained sections and merged to cover the complete cross-sections on which numbers of total fiber and sMHC+ fiber are counted. For macrophage number counting, CD68 positive cells were counted under the microscope. The volume of muscle tissue was determined by measuring the total volume of each section using a stereological, point-counting technique to determine section area and then multiplying that value by the section thickness (10 μm). The numbers of immunolabeled cells in each section were counted and expressed as the number of cells/unit volume of each section.

*Pax7 antibody preparation and immunohistochemistry*

Pax7 hybridoma cells were purchased from Developmental Studies Hybridoma Bank (Iowa City Iowa). Cells were cultured in complete medium consisting of Dulbecco’s Modified Eagle Medium (DMEM) with 1% penicillin- streptomycin (Gibco) and 20% heat-inactivated fetal bovine serum (FBS). Conditioned medium was collected from the cultures and used for purification of antibodies to Pax7, as described previously (Wang *et al.* 2015). The Pax7 antibody was then used for immunohistochemistry staining of cross-sections from quadriceps muscles rapidly frozen in liquid nitrogen-cooled isopentane. Sections were then fixed in 2% paraformaldehyde for 10 minutes and then immersed in antigen retrieval buffer (10 mM sodium citrate, 0.05% Tween 20, pH 6.0) at 95 - 100^o^ C for 40 minutes. Endogenous peroxidase activity in the sectioned tissue was quenched by immersion in 0.3% H_2_O_2_. Sections were then treated with blocking buffer from a mouse-on-mouse immunohistochemistry kit (M.O.M kit; Vector) for 1 hour and immunolabeled with mouse anti-Pax7 antibody overnight at 4^o^ C. Sections were subsequently washed with PBS and then incubated with biotin-conjugated anti-mouse IgG for 30 minutes, followed by incubation for 30 minutes with ABC reagents from the M.O.M kit. Staining was visualized with the AEC kit (Vector). The number of satellite cells/sectioned muscle fiber was determined by counting the number of Pax7+ cells in mid-belly cross-sections of muscles and the total number of fibers per cross-section.

*RNA isolation and quantitative PCR*

Muscles were homogenized in Trizol (Invitrogen) and RNA extracted, isolated and DNase-treated using RNeasy spin columns according to the manufacturer’s protocol (Qiagen). RNA was then electrophoresed on 1.2% agarose gels and RNA quality assessed by determining 28S and 18S ribosomal RNA integrity. Total RNA was reverse transcribed with Super Script Reverse Transcriptase II using oligo dTs to prime extension (Invitrogen) to produce cDNA. The cDNA was used to measure the expression of selected transcripts using SYBR green qPCR master mix according to the manufacturer’s protocol (Bio-Rad). Real-time PCR was performed on an iCycler thermocycler system equipped with iQ5 optical system software (Bio-Rad). Reference genes were chose based following previously described methods (Wang *et al.* 2015). Based on that analysis, RNPS1 and SRP14 were used as reference genes for QPCR experiments with quadriceps muscles. PPIA and TPT1 were used as reference genes for QPCR experiments using myoblasts or bone marrow derived macrophages. The normalization factor for each sample was calculated by geometric averaging of the Ct values of both reference genes using the geNorm software. Primers used for QPCR are listed in Supplemental Table 1.

*Double-labeling for TNFα and CD68*

Frozen, acetone-fixed sections of quadriceps muscles were treated with blocking buffer from M.O.M kit for 1 hour and then immunolabeled with a combination of mouse anti-TNFα (Santa Cruz) and rat anti-CD68 (Biorad) overnight at 4°C. Sections were washed with PBS and then incubated with a combination of Dylight 488 anti-rat IgG and Dylight 594 anti-mouse IgG (1/100; Vector) for 30 minutes in the dark. Sections were then washed with PBS and cover-slipped with Prolong Gold anti-fade reagent with DAPI (Invitrogen).

*Primary myoblast isolation and fusion assay*

Primary myoblasts were isolated following a previously described protocol (Wehling-Henricks *et al.* 2016). Hindlimb and forelimb muscles from 12-month-old mice were removed and rinsed in Dulbecco’s phosphate buffered saline (DPBS). Muscles were minced and digested in 2 ml enzyme buffer (2.4 U/ml dispase, type II (Invitrogen), 1% collagenase, type II (Invitrogen), 2.5 mM CaCl_2_) per mg muscle for 45 minutes at 37˚C with gentle trituration each 15 minutes. The digestate was passed through 70 µm mesh filters and cells were pelleted at 350 x g for 5 minutes. Cells were resuspended in growth medium (Hams F10 (Sigma), 20% FBS (Omega), 2.5 ng/ml bFGF (Sigma), 200 U/ml penicillin and 200 µg/ml streptomycin (Life Technologies)) and pre-plated for 1 hour to remove fibroblasts. Myoblasts were plated on culture dishes coated with 0.01% collagen, type I (Life Technologies) and 2% gelatin and maintained at 37˚C in 5% CO_2_ with medium changes every 3 days. Myoblasts were passed once before seeding for fusion assay.

Myoblasts were seeded in growth medium at 1 x 10^6^ cells/well on 6-well plate with sterile glass coverslips coated as stated above. On day 3 of culture, cells were cultured in Hams F10 media without FBS overnight and then cultured in differentiation medium (Hams F10 (Sigma), 2% horse serum (Omega), 2.5 ng/ml basic fibroblast growth factor (bFGF; Sigma), 200 U/ml penicillin and 200 µg/ml streptomycin (Life Technologies)) for 6 days, with medium changes every 48 hours. Coverslips were then collected and stained with rabbit-anti-desmin (Sigma) with hematoxylin counterstain, using immunohistochemistry protocols described above. For fusion index quantification, the number of myonuclei per myofiber was counted for 500 myofibers randomly sampled throughout the section.

*C2C12 fusion assay with conditioned media from bone marrow-derived macrophages*

BMCs were isolated following a previously described protocol (Wang *et al.* 2015). BMCs were aseptically flushed from femurs and tibias with DPBS (Sigma) and treated with ACK lysing buffer (Gibco, Waltham, MA) to clear red blood cells. Following a DPBS wash and filtration through a 70-µm filter, BMCs were seeded at 5 x 10^6^ per 6-cm dish in RPMI-1640 (Sigma) with 20% heat-inactivated fetal bovine serum (FBS; Omega Scientific, Tarzana, CA), penicillin (100 U/ml; Gibco), streptomycin (100 µg/ml; Gibco) and 10 ng/ml macrophage colony stimulating factor (MCSF; R&D, Minneapolis, MN) at 37˚C with 5% CO_2_ for 6 days. BMDMs were then stimulated for 24-hours with activation media consisting of DMEM with 0.25% heat-inactivated FBS, penicillin, streptomycin and 10 ng/ml MCSF. Conditioned media were collected following activation and spun at 500 x g for 5 minutes to remove floating cells and then frozen at -20⁰ C.

C2C12 cells were obtained from American Type Culture Collection (ATCC). C2C12 cells were seeded at 6 x 10^5^ cells/well on 6-well plate with sterile glass coverslips coated as stated above in DMEM with 10% FBS. Sixteen hours after seeding, seeding medium was removed from culture and cells were washed with DPBS before adding 2 ml / well of BMDM conditioned media. Two days after culture in conditioned media, cells were placed in DMEM only overnight followed by culture in BMDM conditioned media for 5 days, with media changes every 36 hours. Coverslips were collected for desmin staining and fusion index quantification as stated above.

*Bone marrow transplantation and chimerism assay*

BMCs from 2-month-old or 18-month-old wild-type mice were collected as stated above. Recipient *TNFα*-null mice were subjected to myeloablative irradiation prior to bone marrow transplantation. 1 x 10^7^ BMCs were transplanted through tail vein injection following the protocol described previously (Wang *et al.* 2015). Survival of irradiated and transplanted mice is greater than 70% at 20 months of age. Eight months following bone marrow transplantation, muscles and blood were collected from recipient mice. Engraftment of transplanted cells was assessed by fluorescent *in situ* hybridization for X and Y chromosome markers (Kreatech, Leica Labs, Buffalo Grove, IL, USA) in leukocytes isolated from blood collected from each mouse. Isolated leukocytes were adhered to microscope slides, and XY immunolabeled cells and XX immunolabeled cells on each slide were counted. Chimerism was expressed as the number of XX cells/total cell number for each mouse. The percentage of donor derived-cells was greater than 80% for all the animals used in the current investigation.

*Statistics*

Data are presented as mean ± sem. One-way analysis of variance was used to test whether differences between 3 or more groups were significant at p < 0.05. Significant differences between groups were identified using Tukey’s Post Hoc test. Comparisons of two groups of values were analyzed using the unpaired, two-tailed t test.
